# To a Young Doctor, from His Father, (A Homœopathic Physician)

**Published:** 1878-11

**Authors:** 


### To a Young Doctor from his Father.—(A Homoeopathic Physician.)

My Dear Son:—Your letter of inquiry as to a "policy" which shall serve you as a guide in your professional life, has been received. I rejoice at your beginning so early in life to use the term "policy." Never lose sight of it, and it will guide you to social and professional eminence, as it has your father before you.

Your entering upon professional life at a time when our school is so greatly agitated and seems in danger of rupture, requires, as you indeed intimated, an unusual amount of caution. Could I foresee which side would win in the end, my advice would be short and to the point. But I myself am sorely perplexed and find it trying, at my time of life, to accommodate myself to the needs of the hour. I have even thought of retiring from active work, but my position is too prominent. I have only lately been elected an honorary member of the —— State Society, am president of the —— Central Society, chairman of the Bureau on Dose of the —— Medical Institute and my name may possibly be used (without my sanction, of course) as a candidate for a chair in the —— State University. (By the bye Dr. B— has promised to help me in this little matter and his influence is very powerful). All this tempts me to stick, hoping, that no matter who wins I shall be on the right side.

As to yourself, I trust that you will ever remember that to insure success you must be prepared to throw overboard all notions of so-called consistency. A young man of talent can not afford to sacrifice position to a mere whim. It will seldom pay you to fight the majority. Act and fraternize with them. But while doing so, conciliate by a show of sympathy to a minority, if composed of men of ability, who are apt to be in the right, but have not the tact to accommodate themselves to others, and thus usually succeed in only bringing upon themselves disfavor and contempt. I might name such men; but your acquaintance with the current literature of our school makes illustrating unnecessary. On questions of importance and of interest never commit yourself if you can avoid it. An easy way of doing this, is always to agree with present company. To give an example: No one but a fool would talk anything but high-dilutionism to Dr. L—; in conversation with a man like Dr. H— of E— it would be thoughtless to omit a shrug of the shoulders or a confidential smile while mentioning them. "Individualize" with one man; but "generalize" with his neighbor.

To do this successfully be shrewd and bold. I know a man who read a paper on "Pure Homœopathy" at a meeting of medical men, deploring mongrelism and especially the use of crude drugs. He had his case with him, (thoughtlessness, mind you). A mischief loving listener opened it while its owner was reading his high toned paper, and upon finding in it *Calomel*, *Quinine*, *Dover's Powders*, *Ipecac*, etc., all of them in crude, exhibited them good naturedly and thus absolutely ruined the effects of a carefully prepared paper. There you had a man who was sensible on the point of medication—but he should have left his case at home. I felt sorry to see a man of his ability overwhelmed with ridicule, owing to a little carelessness. Take due warning, my son. Nothing would so mortify me as the thought that you might be guilty of similar imprudence.

I need not speak to you of high dilutions in your practice, for you are nearly twenty-five years old. I have taught you the use of medicines in such doses as would produce desirable and appreciable effects. You have been shown how to discriminate between true science and blind enthusiasm. Remember these lessons! Hahneman was a man of originality and respectability, but you are too well read to follow him blindly. Men of his eccentricity are not often safe leaders. While it then seems necessary to avow a great deal of reverence for the "master" as an eminent man of the day has it; while it will do well enough at times to out Hahnemann Hahnemann himself in lofty enthusiasm for the "cause" and all that, remember that the fluid extract of a drug is much safer to depend upon than the two thousandth dilution. As you come across reports of cures with Swan's high potencies, think of "*skimmed milk*." But it would not be a bad move, could you occasionally publish a cure with the millionth potency, while showing a general disposition to favor low potencies. You will, by doing so, get the reputation of a careful, earnest, thoughtful and candid laborer in the field and may become a man of note, as others have by the use of the same means.

Time however hurries. Farewell for to-day. Remember, that I will advise you at all times to your advantage. Do not fail to make good the hopes of my heart, centered in you. Above all, my son, take no sides at present. Blows are falling rapidly and dealt carelessly. Take heed not to put yourself in their way.

Your co-laborer in the cause of Homœopathy and affectionate father, H. R. A.

*Cincinnati Advance*, (*Homœopathic Journal.*)

